# Optimization of high-intensity resistance exercise protocols for improving bone mineral density in the elderly without chronic diseases: a systematic review and network meta-analysis

**DOI:** 10.3389/fphys.2025.1589200

**Published:** 2025-06-10

**Authors:** Yang Cheng, Yue Yi, Shumin Bo, Jun Mao, Jing Ma

**Affiliations:** ^1^ School of Life Science, Beijing Institute of Technology, Beijing, China; ^2^ School of Kinesiology and Health, Capital University of Physical Education and Sports, Beijing, China; ^3^ School of Physical Education, Jiangsu University of Technology, Changzhou, Jiangsu, China

**Keywords:** resistance exercise, resistance training, strength, elderly, bone mineral density, osteopenia, network, meta-analysis

## Abstract

**Objective:**

This study aims to explore the effects of high-intensity resistance exercise (HIRE) protocols on improving bone mineral density (BMD) in the elderly without chronic diseases by using a forest plot and network meta-analysis.

**Methods:**

A systematic search was performed across seven databases including PubMed, Web of Science, Embase, Cochrane Library, China National Knowledge Infrastructure, Wan Fang and VIP, to investigate the effects of HIRE on BMD in the elderly by December 9, 2024. The search strategy incorporated Boolean operators (AND, OR, NOT) to refine the selection of relevant studies. The methodological quality was assessed by using Risk of Bias tool 2 and Tool for the Assessment of Study Quality and Reporting in Exercise, and data analysis was performed using Stata 17.

**Results:**

A total of 13 RCTs involving 616 participants were included. Among the various HIRE protocols, two demonstrated positive effects on lumbar spine and whole-body BMD, while four were positive in improving femoral neck BMD compared to the control group. The network meta-analysis revealed that 3M(9–10) was more effective than 2M(6–8), 3M(6–8) and 1M(6–8) in enhancing lumbar spine BMD. However, no significant differences were observed among the HIRE protocols for femoral neck and whole body BMD. According to the SUCRA rankings, 3M(9–10) was the most effective protocol for improving lumbar spine (94.7%) and femoral neck BMD (82.3%), while 2M(9–10) ranked highest for whole body BMD improvement (61.6%).

**Conclusion:**

HIRE protocol is critical to ensure BMD improvement for elderly without chronic diseases, and not all HIRE protocols yield positive effects on BMD. Compared to other sites, lumbar spine BMD appears to be more responsive to HIRE. A 2–3 times, multiple-set regimen may be more beneficial for improving lumbar spine, whole-body, and femoral neck BMD in the elderly, and performing 9–10 repetitions per set is particularly important for improving BMD in older adults.

**Systematic review registration number:**

https://www.crd.york.ac.uk/PROSPERO/, identifier registration number. CRD42024543517.

## 1 Introduction

The global elderly population is rapidly increasing, and the issue of population aging is becoming increasingly severe (Sun et al., 2024). Among a series of health risks faced by the elderly, the most significant is the risk of fractures (Myeroff et al., 2018), a common complication of osteoporosis (LeBoff et al., 2022). Osteoporosis primarily results from decrease in bone mineral density (BMD) during aging (Henriksen et al., 2007; Kassem et al., 1997), which occurs due to an imbalance in bone remodeling, where bone resorption exceeds bone formation (Fan et al., 2024). As BMD decreases, bone mass is lost, structural integrity weakens, and the mechanical strength and elasticity of bones are reduced, significantly increasing the risk of osteoporosis and fractures (Osterhoff et al., 2016). Therefore, early prevention aimed at mitigating BMD reduction are critical to reduce the incidence of osteoporosis and lower fracture risks, preserving the health of elderly individuals (Bhatnagar and Kekatpure, 2022; Brooke-Wavell et al., 2022).

So far, prevention for BMD reduction primarily includes pharmacological treatments and exercise interventions. Pharmacological treatments, such as the use of alendronate (Kendler et al., 2020), is capable to improve BMD by inhibiting bone resorption. Despite of being effective, this approach has some limitations, including high costs, drug dependence, and potential negative effects on health (e.g., allergies, tumors) (Adams et al., 2021; De Martinis et al., 2020). Exercise provides an alternative method with low costs. Furthermore, common exercise hardly destroys normal metabolism, with fewer risks of negative effects (Aibar-Almazán et al., 2022). It is worth noting that the exercise-induced improvements in BMD exhibit site-specific effects, which are influenced by the type of exercise and the mechanical load applied to the bone (Marques et al., 2011). Therefore, numerous previous studies have shown that resistance exercise is more effective than other forms of exercise in improving BMD (Hong and Kim, 2018; Lambert et al., 2020). Notably, the intensity of resistance exercise is a critical factor, with previous studies showing that high-intensity resistance exercise (HIRE) significantly improves BMD compared to low-intensity resistance exercise (Vincent and Braith, 2002). The main reason may be that HIRE provides sufficient mechanical stress to stimulate bone remodeling (Bemben et al., 2000). Consequently, HIRE is promising for the early prevention of BMD reduction in the elderly.

However, HIRE introduces challenges and risks for elderly individuals, as they are more vulnerable to the effects of exercise, and the metabolic strain of excessive exercise may result in muscle loss and even bone microstructural damage (Hernández-Álvarez et al., 2023; Kibler et al., 1992). In contrast, although reducing load decrease the risk of overtraining and improve safety, low-load may not provide sufficient mechanical load to effectively stimulate bone remodeling, thereby limiting the improvement in BMD (Robling and Turner, 2009). Therefore, HIRE protocol optimization is of significant importance. However, only a few study has directly compared the effects of HIRE frequency on BMD improvement in the elderly (Taaffe et al., 1999). HIRE protocol involves a series of training parameters, and other parameters of HIRE, such as number of sets and repetitions, have not been systematically explored.

Currently, extensive research has been conducted on resistance exercise interventions for older adults with chronic conditions, such as osteopenia and osteoporosis (Cui et al., 2023; Linhares et al., 2022; Zhang et al., 2022). However, due to inherent differences in physiological and health characteristics among various populations, these intervention protocols may not be directly applicable to older adults without chronic diseases (Collins, 1999). Thus, it is imperative to further investigate and develop tailored resistance exercise strategies to optimize health outcomes for this distinct demographic, improve bone health, and better prevent bone-related diseases. Recently, a series of studies have demonstrated the validity of meta-analysis in investigating the relationship between resistance exercise and BMD in older adults (Kitsuda et al., 2021; Souza et al., 2020). For instance, Wang et al. utilized network meta-analysis to assess the effects of resistance training on BMD in postmenopausal women (Wang et al., 2023). Similarly, other studies have confirmed the feasibility of using meta-analysis to evaluate the impact of different training protocols on BMD improvement (Florence et al., 2023; Mohebbi et al., 2023). These studies highlight the importance of resistance exercise in mitigating age-related bone loss, enhancing skeletal strength, and reducing osteoporosis risk.

However, these studies do not focus on the crucial issue of resistance exercise, specifically exercise intensity. Given the existing research on HIRE, it is reasonable to hypothesize that under high-intensity conditions, different resistance exercise protocols may have varying effects on BMD in older adults. Therefore, this study conducted a network meta-analysis to optimize HIRE protocols for improving BMD in the elderly without chronic diseases. First, relevant studies which examined the effects of HIRE on BMD in the elderly without chronic diseases were selected. Then, the effects of various HIRE protocols on BMD improvement were analyzed by using forest plots compared to the control group. Finally, network meta-analysis combined with surface under the cumulative ranking curve (SUCRA) was used to determine the optimal HIRE protocols for BMD improvement.

## 2 Materials and methods

This meta-analysis has been registered in the International Prospective Register of Systematic Reviews (registration number: CRD42024543517), and follows the Preferred Reporting Items for Systematic Reviews and Meta-Analyses (PRISMA) statement for network meta-analyses (Page et al., 2021). A complete PRISMA checklist can be found in Supplementary Table S1.

### 2.1 Search strategy

As of December 9, 2024, a systematic search was conducted in four English-language databases (PubMed, Web of Science, Embase, and the Cochrane Library) and three Chinese-language databases (China National Knowledge Infrastructure (CNKI), Wan Fang, and VIP) to identify relevant RCTs investigating the effects of HIRE on BMD in older adults, using Boolean operators (AND, OR, NOT). The search terms used included: “elderly,” “resistance,” “resistance training,” “resistance exercise,” “strength,” “strength training,” “strength exercise,” “bone mineral density,” and “BMD.” The search strategy for PubMed is shown in Table 1. Additionally, the detailed search strategy is provided in the Supplementary Table S2. Furthermore, we also searched specific websites and screened citations from relevant articles to ensure a more comprehensive selection of studies.

**TABLE 1:** The search strategy for PubMed.

Query	Search terms
#1	(((((((((“Aged” [Mesh]) OR (Aging Women [Title/Abstract])) OR (Aging Men [Title/Abstract])) OR (Elderly [Title/Abstract])) OR (Older adults [Title/Abstract])) OR (Older adult [Title/Abstract])) OR (Older Women [Title/Abstract])) OR (Older Men [Title/Abstract])) OR (Frail Elderly [Title/Abstract])) OR (Postmenopausal Women [Title/Abstract])
#2	(((((((“Bone Density” [Mesh]) OR (Bone Densities [Title/Abstract])) OR (Density, Bone [Title/Abstract])) OR (Bone Mineral Density [Title/Abstract])) OR (Bone Mineral Densities [Title/Abstract])) OR (Density, Bone Mineral [Title/Abstract])) OR (Bone Mineral Content [Title/Abstract])) OR (Bone Mineral Contents [Title/Abstract])
#3	(((((((((“Resistance Training” [Mesh]) OR (Resistance exercise [Title/Abstract])) OR (Training, Resistance [Title/Abstract])) OR (Strength Training [Title/Abstract])) OR (Training, Strength [Title/Abstract])) OR (Strength exercise [Title/Abstract])) OR (Progressive resistance training [Title/Abstract])) OR (Weight training [Title/Abstract])) OR (Weight exercise [Title/Abstract])) OR (Weight lift [Title/Abstract])
#4	#1 AND #2 AND #3

### 2.2 Eligibility criteria

#### 2.2.1 Inclusion criteria

(1) Study Population: Older adults aged 55 and above (Massini et al., 2022), free from chronic diseases, and without a regular exercise habit in the past 6 months.
(2) Intervention: HIRE performed at or above 80% of one repetition maximum (1RM). The resistance exercise loads reported as repetition maximum (RM) were converted to a percentage of %1RM using the equation (Currier et al., 2023):

$$\%1RM=100-\left(RM\times 2.5\right)$$

(3) Control Group: Control groups consisted of either non-exercise participants.
(4) Outcome Measures: BMD of the lumbar spine, whole body, and femoral neck, measured using dual-energy X-ray absorptiometry.
(5) Study Type: Randomized controlled trials (RCTs).

#### 2.2.2 Exclusion criteria

Studies were excluded that involved participants with chronic diseases affecting bone metabolism, such as osteoporosis, osteopenia, diabetes, heart disease, or sarcopenia. Additionally, studies that did not employ resistance exercise as the intervention, did not specify resistance intensity, or used an alternative intensity descriptor besides 1RM were excluded. Furthermore, studies involving moderate or low-intensity resistance exercise (i.e., <80% of 1RM), non-randomized controlled trials, self-controlled trials, animal studies, duplicate publications, reviews, and conference abstracts were also excluded.

### 2.3 Study selection and data extraction

After removing duplicates using EndNote (20.6, Clarivate Co., United States), two authors independently screened the titles and abstracts based on inclusion and exclusion criteria to identify potentially eligible studies. The full texts were then reviewed to confirm the final included studies. Data were extracted by the two authors using Microsoft Office Excel, including the first author, publication year, information of participants, resistance exercise parameters, and outcome measures. Any discrepancies in study selection or data extraction were resolved through discussion with a third author.

### 2.4 Methodological quality assessment of included studies

The risk of bias in the included studies was assessed using the Cochrane Risk of Bias tool 2 (Rob2), which includes: (1) bias arising from the randomization process, (2) deviations from the intended interventions, (3) missing outcome data, (4) measurement of the outcomes, and (5) selective reporting of results (Sterne et al., 2019). Based on these five domains, studies were classified into three categories: low risk, some concerns, or high risk of bias. Furthermore, to ensure a more comprehensive quality assessment, we further employed the Tool for the Assessment of Study Quality and Reporting in Exercise (TESTEX). This validated tool, with a maximum score of 15, evaluates study quality across 12 key domains, providing an in-depth assessment of methodological rigor and reporting standards in exercise-based research (Smart et al., 2015).

### 2.5 Statistical analysis

The forest plot and network meta-analysis were performed using Stata (version of 17, StataCorp LLC, United States) (Antoniou et al., 2019; Shim et al., 2017). Considering the different measurement units in the outcome assessments, the standardized mean difference (SMD) and its 95% confidence intervals (95% CI), expressed as Hedges’ g because it corrects for SMD for small samples (Taylor and Alanazi, 2023), were calculated using either a random-effects or fixed-effects model. Heterogeneity was rigorously assessed through the I^2^ test, where the I^2^ values were categorized to determine the extent of heterogeneity, with values of 25%, 50%, and 75% representing low, moderate, and high heterogeneity, respectively. When the I^2^ value equals or exceeds 50%, substantial heterogeneity is suggested, thus a random-effects model is applied. Conversely, when the I^2^ value is below 50%, a fixed-effects model is used (Higgins et al., 2003). An effect size of approximately 0.4 indicates a small effect, around 0.4 to 0.7 signifies a moderate effect, and greater than 0.8 represents a significant effect (Kons et al., 2023). A network geometry diagram was constructed to visualize the relationship between different HIRE protocols and the control group (Chaimani et al., 2013). In instances where the network contained open loops, a consistency model was employed; conversely, loop inconsistency was tested to assess the consistency of the outcome measures. If *P* > 0.05, indicating good consistency between direct and indirect evidence, a consistency model was adopted. Otherwise, subgroup and regression analyses were conducted to explore sources of heterogeneity. The SUCRA plots were generated to identify the most effective HIRE protocol, and comparison-adjusted funnel plots were utilized to detect publication bias and small study effects (Higgins et al., 2012). In funnel plots, the x-axis represents the adjusted effect size, which indicates the deviation of each study’s effect size from the overall effect for a specific comparison, while the Y-axis (Standard error (SE) of effect size) represents the SE of the effect size (if the number of studies is fewer than 10, this will be addressed in the discussion section). The reliability of the study results was independently assessed by two authors using the Grading of Recommendations Assessment, Development, and Evaluation (GRADE) system (Dahm et al., 2024). Initially, all included studies were RCTs, and therefore were rated as high quality. However, the reliability of the study results was downgraded due to the influence of several factors, including risk of bias, inconsistency, indirectness, imprecision, and other relevant considerations. Consequently, the final assessment of reliability was categorized into four levels: high, moderate, low, and very low.

## 3 Results

### 3.1 Literature search and screening

**FIGURE 1:**
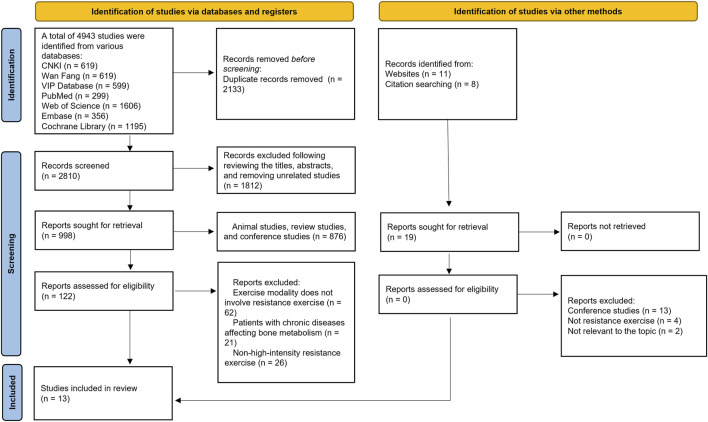
Literature screening process.

Figure 1 presents the detailed literature search process, through which 13 studies meeting the inclusion criteria were selected (Armamento-Villareal et al., 2020; Bemben et al., 2000; Bemben et al., 2010; Bocalini et al., 2009; Chuin et al., 2009; Kerr et al., 1996; Marques et al., 2011; Nelson et al., 1994; Taaffe et al., 1999; Verschueren et al., 2004; Vincent and Braith, 2002; Whiteford et al., 2010; Xiang and Cizhong, 2017).

### 3.2 Basic information of included studies and quality assessment

**FIGURE 2:**
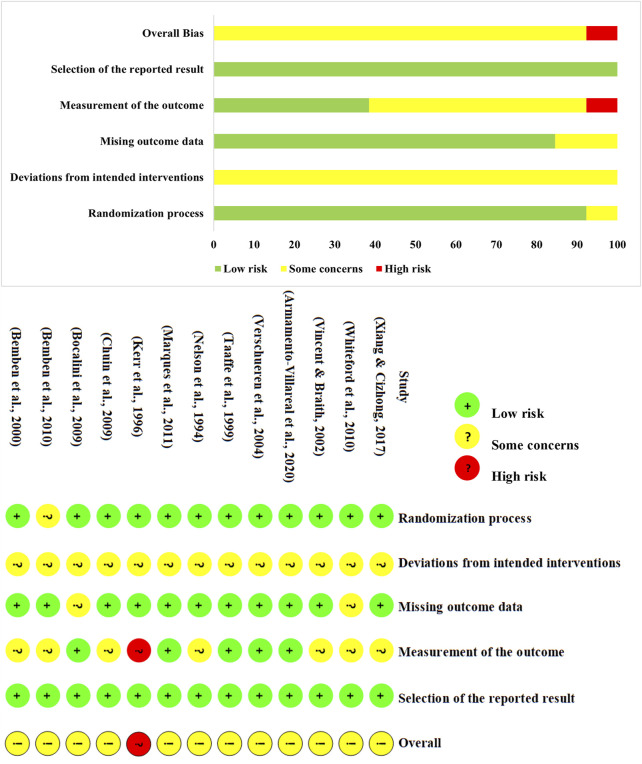
The quality assessment of the included studies.

Among the 13 studies, 616 participants were involved. Detailed characteristics of the 13 studies are presented in Table 2. To maintain homogeneity within the study population, none of the participants were taking supplements that could potentially influence BMD, such as vitamin D or calcium. Furthermore, female participants did not undergo hormone replacement therapy. The average adherence rate of the participants was 85.7%, indicating a relatively high level of compliance with the intervention protocol (Brobakken et al., 2022). The risk of bias assessment using the RoB2 tool is presented in Figure 2. Regarding randomization, all studies were rated as “low risk”, except for one study, which lacked a clear description of the randomization process. For deviations from the intended intervention, given the nature of exercise interventions, conducting a double-blind randomized controlled trial is inherently challenging. Consequently, all studies were rated as “some concerns” in this domain. For missing outcome data, 2 studies were rated as “some concerns” due to missing data that could potentially impact the results. Regarding outcome measurement, 7 studies were rated as “some concerns”. Additionally, Kerr et al. reported adverse events that could have influenced the results, therefore being rated as high risk (Kerr et al., 1996). Lastly, no evidence of selective reporting bias was identified in any of the included studies. Overall, the inherent challenges in implementing double-blind exercise interventions led to a relatively lower methodological quality among the included studies. The quality assessment of the included studies using the TESTEX is presented in Table 3.

**TABLE 2:** The basic characteristics of included studies.

Author/Year	Country	Samples	Duration	Mean Ages	Body Mass(kg)	BMI	Intensity	Frequency	Protocol	Sets × Reps	Intermittent Time	BMD Outcome	Pre-Ex (g/cm^2^)	Post-Ex (g/cm^2^)
Nelson et al. (1994)	USA	20	1 year	61.1 ± 3.7	64.7 ± 7.7	24.4 ± 2.5	The adaptation phase: 50%–60% 1RM, followed by increase to 80%	2	Hip extension, knee extension, and lateral pulldown using pneumatic resistance machines, with Borg scale level 16 for back extension and abdminal flexion	3 × 8	90–120 s	FN	0.853 ± 0.134	∼0.858
												LS	1.020 ± 0.164	∼1.029
Taaffe et al. (1999)	USA	11	24 weeks	68.5 ± 3.6	70.2 ± 14.4	23.9 ± 2.8	The adaptation phase: 60% 1RM, followed by increase to 80%	1	Eight exercises (bench press, military press, latissimus pull-down, biceps curl, leg press, knee flexion/extension and back extension)	3 × 8	N	LS	0.960 ± 0.192	1.025 ± 0.006
		12		69.4 ± 3.0	70.3 ± 8.9	22.8 ± 2.1		2					1.084 ± 0.262	1.033 ± 0.006
		11		71.0 ± 4.1	72.4 ± 13.0	24.2 ± 1.9		3					0.999 ± 0.152	1.032 ± 0.007
Bemben et al. (2000)	USA	10	6 months	50.5 ± 2.0	74.7 ± 5.6	28.7 ± 2.4	80% 1RM	3	Twelve exercises (quadriceps extension, hamstring flexion, leg press, shoulder press, biceps curl, triceps extension, seated row, latissimus pull, hip extension, hip flexion, hip abduction, hip adduction)	3 × 8	N	WB	1.154 ± 0.027	1.141 ± 0.027
												FN	0.910 ± 0.048	0.903 ± 0.045
												LS	1.125 ± 0.044	1.116 ± 0.043
Vincent and Braith (2002)	USA	22	6 months	66.6 ± 7	74.8 ± 15	N	80% 1RM	3	resistance training machines (abdominal crunch, leg press, leg extension, leg curl, calf press, seated row, chest press, overhead press, biceps curl, seated dip, leg abduction, leg adduction, lumbar extensions)	1 × 8	120 s	WB	1.192 ± 0.100	1.182 ± 0.100
												FN	0.852 ± 0.100	0.869 ± 0.100
Verschueren et al. (2004)	Belgium	22	6 months	63.9 ± 3.8	70.47 ± 9.6	27.4 ± 3.5	The adaptation phase: 20RM followed by increase to 8RM	3	Knee extension and leg press exercises	1 × 8/3 × 12	N	WB	1.016 ± 0.078	1.016 ± 0.077
												LS	0.900 ± 0.136	0.901 ± 0.135
Chuin et al. (2009)	Canada	11	6 months	65.4 ± 3.5	66.61 ± 8.47	26.54 ± 2.74	80% 1RM	3	leg press, bench press, leg extension, shoulder press, sit-up, seated row, triceps extension, biceps curl	3 × 8	90–120 s	FN	0.910 ± 0.08	0.910 ± 0.08
												LS	1.06 ± 0.13	1.06 ± 0.16
Bemben et al. (2010)	USA	22	8 months	64 ± 0.9	76.6 ± 3.16	30 ± 1	80% 1RM	3	supine two-leg press, hip flexion, hip extension, hip abduction, hip adduction, seated military press, lat pull down, seated row	3 × 10	N	WB	1.15 ± 0.021	1.149 ± 0.022
												FN	0.902 ± 0.021	0.898 ± 0.021
												LS	1.163 ± 0.028	1.156 ± 0.030
Whiteford et al. (2010)	Australia	73	1 year	64 ± 6	82.4 ± 10.7	26.4 ± 3.1	The adaptation phase: minimal resistance followed by increase to 80% 1RM	3	Hip flexion, hip extension, hip abduction, hip adduction, calf raise, tricep pushdown, wrist curl, reverse wrist curl, bicep curl, forearm pronation, forearm supination	3 × 8	N	WB	1.231 ± 0.110	1.230 ± 0.108
												FN	0.966 ± 0.142	0.969 ± 0.140
												LS	1.236 ± 0.189	1.235 ± 0.185
Marques et al. (2011)	Portugal	23	8 months	67.3 ± 5.2	N	28.8 ± 4.6	The adaptation phase: 50%–60% 1RM followed by increase to 75%–80% 1RM	3	Leg press, leg extension, seated leg curl, hip abduction, double chest press, lateral raise, overhead press, abdominal wall	2 × 6–8	120 s	FN	0.684 ± 0.082	0.676 ± 0.090
Armamento-Villareal et al. (2020)	USA	40	6 months	70 ± 5	101.7 ± 18.2	36.7 ± 5.8	The adaptation phase: 65% 1RM followed by increase to 85% 1RM	3	Nine upper and lower body exercises using weight lifting machines at 85% 1RM	2–3 × 8–12	N	WB	1.115 ± 0.021	∼1.12
												FN	0.804 ± 0.02	∼0.801
												LS	1.144 ± 0.033	∼1.152
Xiang and Cizhong (2017)	China	13	24 weeks	62.3 ± 3.6	62.9 ± 14	23.7 ± 2.1	80% 1RM	2	quadriceps, hamstrings, pectoralis major, upper and lower back, triceps, biceps, core muscles	2 × 8–10	N	WB	1.104 ± 0.097	∼1.113
												LS	1.082 ± 0.186	∼1.091
Bocalini et al. (2009)	Brazil	15	24 weeks	69 ± 9	68 ± 6	28 ± 4	The adaptation phase: 50% 1RM, followed by increase to 85%	3	leg press, chest press, leg curl, latissimus pull down, elbow flexion, elbow extension, leg extension, upper back row, military press, hip abductor, hip adductor, abdominal curls	3 × 10	60 s	FN	0.705 ± 0.001	0.704 ± 0.001
												LS	0.881 ± 0.001	0.880 ± 0.001
Kerr et al. (1996)	Australia	28	1 year	58.4 ± 3.7	69.4 ± 11.4	N	8RM	3	upper limb: biceps curl, wrist curl, reverse wrist curl, triceps extension, forearm pronation, forearm supination; lower limb: leg press, hip abduction, hip adduction, hamstring curl, hip flexion, hip extension	3 × 8	120–180 s	FN	0.72 ± 0.10	∼0.72

BMD, bone mineral density; BMI, body mass index; Ex, exercise; FN, femoral neck; LS, lumbar spine; WB, whole body; 1RM, one repetition maximum; RM, repetition maximum; Reps, repetitions.

**TABLE 3:** TESTEX scale for assessing study quality.

Author/Year	1	2	3	4	5	6	7	8	9	10	11	12	Overall
Nelson et al. (1994)	1	1	0	1	0	3	1	2	1	1	1	1	13
Taaffe et al. (1999)	1	1	0	1	0	2	0	2	1	0	1	1	10
Bemben et al. (2000)	1	1	0	1	0	2	0	2	1	0	1	1	10
Vincent and Braith (2002)	1	1	0	1	0	1	0	2	1	1	1	1	10
Verschueren et al. (2004)	1	1	0	1	0	2	0	2	1	1	1	1	11
Chuin et al. (2009)	1	1	0	1	0	1	0	2	1	0	1	1	9
Bemben et al. (2010)	1	0	0	1	0	2	0	2	1	0	1	1	9
Whiteford et al. (2010)	1	1	0	1	0	2	1	2	1	1	1	1	12
Marques et al. (2011)	1	1	0	1	0	1	1	2	1	1	1	1	11
Armamento-Villareal et al. (2020)	1	1	0	1	1	2	1	2	1	0	1	1	12
Xiang and Cizhong (2017)	1	1	0	1	0	0	0	2	1	0	1	1	8
Bocalini et al. (2009)	1	1	0	1	1	1	0	2	1	0	1	1	10
Kerr et al. (1996)	1	1	0	1	0	2	0	2	1	0	1	1	10

1, Eligibility criteria specified; 2, Randomization specified; 3, Allocation concealment; 4, Groups similar at baseline; 5, Blinding of assessor; 6, Outcome measures assessed in 85% of patients; 7, Intention-to-treat analysis; 8, Between-group statistical comparisons reported; 9, Point measures and measures of variability for all reported outcome measures; 10, Activity monitoring in control groups; 11, Relative exercise intensity remained constant; 12, Exercise volume and energy expenditure.

### 3.3 Meta-analysis

#### 3.3.1 Network geometry

**FIGURE 3:**
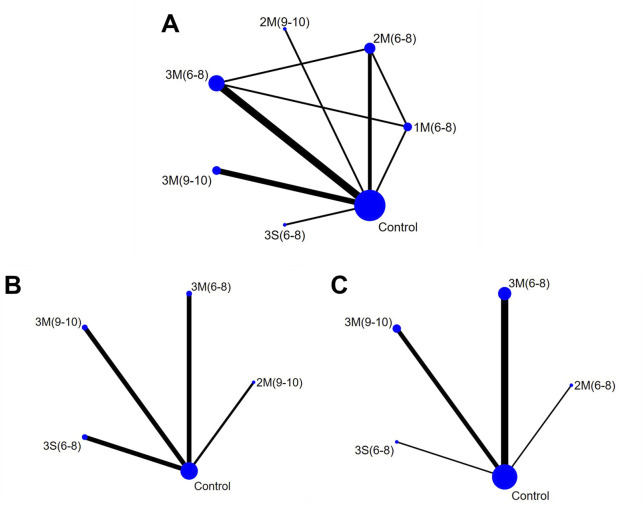
Network Geometry Diagram **(A)** Lumbar Spine BMD; **(B)** Whole-body BMD; **(C)** Femoral Neck BMD). 1M(6–8): once per week, multiple sets, 6-8 reps; 2M(6–8): twice per week, multiple sets, 6-8 reps; 2M(9–10): twice per week, multiple sets, 9–10 reps; 3M(6–8): three times per week, multiple sets, 6-8 reps; 3M(9–10): three times per week, multiple sets, 9–10 reps; 3S (6–8): three times per week, single set, 6-8 reps; Control: no exercise intervention.

The network geometry diagrams are shown in Figure 3, in which each node represents a protocol with specific HIRE parameters and each line indicates a direct comparison. Protocol definition is derived from the selection of HIRE parameters, including frequency (3/2/1), single (S) or multiple (M) sets (Currier et al., 2023), and repetitions per set (6–8) and (9–10). If repetitions are given as a range, their average value is used for representation. All the HIRE protocols were compared with control, while no direct comparison between different HIRE protocols was existed in the network geometry of whole-body and femoral neck BMD. In contrast, pairwise comparison was performed among 1M(6–8), 2M(6–8) and 3M(6–8) in the lumbar spine network geometry.

#### 3.3.2 Comparison between HIRE and control groups

**FIGURE 4:**
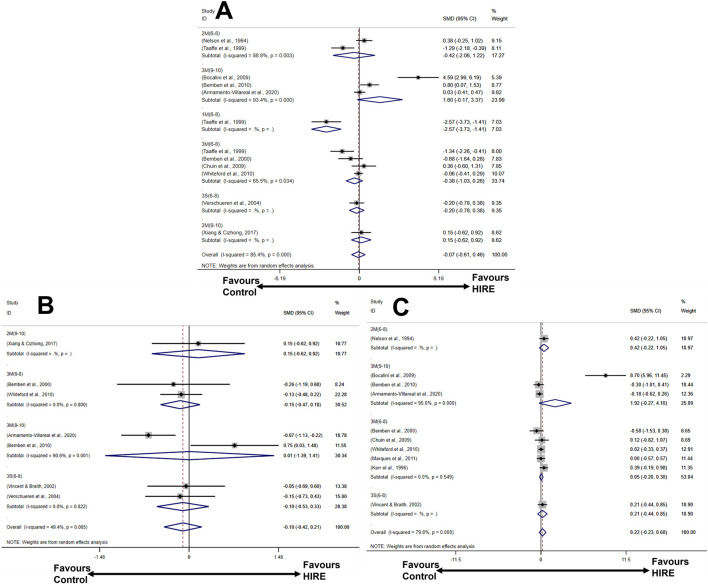
Forest plot illustrating the effects of HIRE on BMD improvement in elderly without chronic diseases compared to the control group **(A)** Lumbar spine BMD; **(B)** Whole-body BMD; **(C)** Femoral neck BMD).

The forest plot provides a visual representation of the overall effects of HIRE protocols on BMD (Figure 4). For lumbar spine BMD, six HIRE protocols were analyzed across ten studies, with effect sizes pooled using a random-effects model. The results indicated that only 3M(9–10) (large effect size, SMD 1.60, 95% CI [−0.17, 3.37]) showed a certain degree of improvement, although it did not reach statistical significance. Regarding whole-body BMD, a random-effects model was applied for meta-analysis. The findings demonstrated that 2M(9–10) (small effect size, SMD 0.15, 95% CI [−0.62, 0.92]) exhibited a potential positive effect on BMD; however, its 95% confidence intervals crossed the null line. Finally, for femoral neck BMD, effect sizes were pooled using a random-effects model, revealing that 2M(6–8), 3M(9–10), 3M(6–8), and 3S (6–8) contributed to varying degrees of BMD improvement.

#### 3.3.3 Network meta-analysis of different HIRE protocols

**FIGURE 5:**
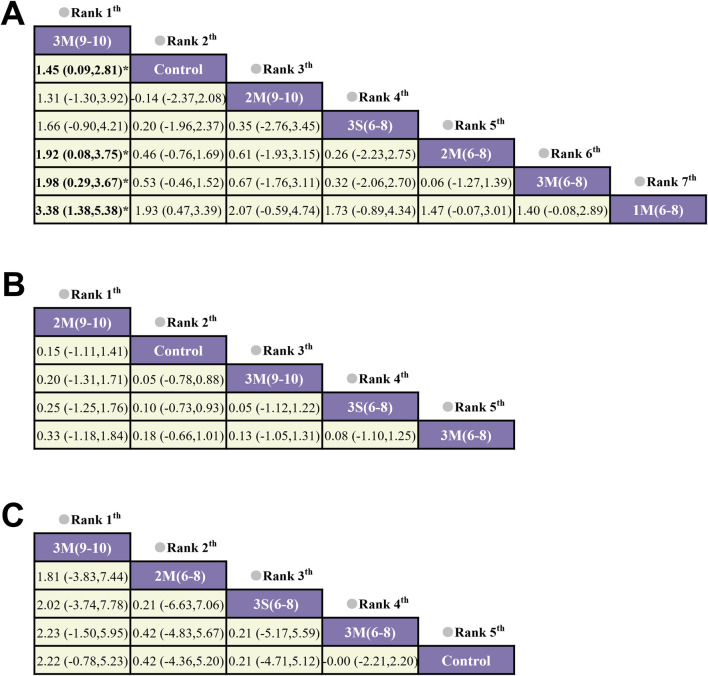
The network meta-analysis on the effects of different HIRE protocols on BMD in elderly without chronic diseases **(A)** Lumbar spine BMD; **(B)** Whole-body BMD; **(C)** Femoral neck BMD).

**FIGURE 6:**
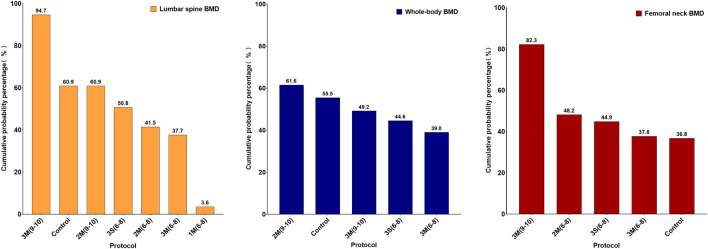
Ranking of SUCRA for different HIRE protocols (Left: Lumbar spine BMD; Middle: Whole-body BMD; Right: Femoral neck BMD).

The results of the loop inconsistency test indicated that the direct and indirect evidence demonstrated satisfactory consistency (p > 0.05), suggesting that there were no significant discrepancies between them within the closed loops. Therefore, a consistency model was employed for the analysis. For lumbar spine BMD, the results showed that 3M(9–10) was more effective in improving BMD than 2M(6–8), 3M(6–8) and 1M(6–8), with a statistically significant difference (p < 0.05) (Figure 5). However, for whole body and femoral neck BMD, no significant statistical differences were observed between the various HIRE protocols. Subsequently, the effectiveness ranking of different HIRE protocols was presented by comparing SUCRA scores (Figure 6). A higher SUCRA value (ranging from 0% to 100%) indicates a more favorable effect of HIRE on improving BMD. The results indicated that 3M(9–10) was the most effective protocol for improving lumbar spine and femoral neck BMD, while 2M(9–10) was the most effective protocol for improving whole body BMD.

#### 3.3.4 Publication bias

**FIGURE 7:**
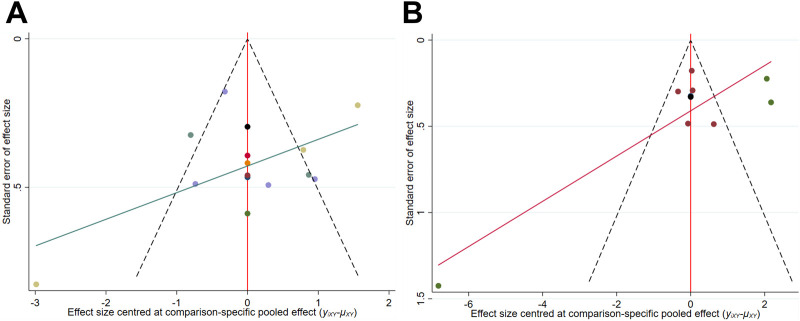
Comparison-adjusted funnel plot **(A)** Lumbar spine BMD; **(B)** Femoral neck BMD.

As shown in Figure 7, for lumbar spine and femoral neck BMD, although the majority of studies fall within the funnel plot’s confidence boundaries, the distribution of a few studies indicates a potential presence of slight publication bias. Since fewer than 10 studies were included for whole body BMD, a funnel plot was not generated. The GRADE assessment indicates that the certainty of the evidence has been downgraded to low due to the risk of bias and the small sample size (Supplementary Table S3).

## 4 Discussions

This study utilized forest plot and network meta-analysis to investigate the impacts of HIRE protocols on improving BMD in elderly without chronic diseases. In the forest plot, approximately fewer than half of HIRE protocols had SMD values of more than 0 in lumbar spine and whole-body BMD improvement, but the CIs of all these protocols crossed null line. This phenomenon suggested that the effectiveness of HIRE in improving lumbar spine and whole-body BMD remains further explore. Notably, most HIRE protocols showed an SMD less than 0, highlighting the potential risks of resistance training, particularly in the context of high-intensity (Garber et al., 2011).Therefore, it is necessary to further discuss the intensity of HIRE. According to the “bone-muscle interaction” theory, the mechanical forces generated by muscle contraction are transmitted to the bone via tendons, thereby promoting bone remodeling (Hao et al., 2024). Increasing exercise intensity appropriately can further enhance BMD, stimulating adaptive responses in bone tissue, promoting mineral deposition, and improving bone structural stability. However, excessive intensity may surpass an individual’s adaptive capacity, potentially leading to stress fractures or other musculoskeletal issues (Hamstra-Wright et al., 2021). Therefore, optimizing HIRE protocol is essential for maximizing its osteogenic benefits while minimizing potential risks.

Previous studies have demonstrated the positive effects of HIRE on BMD. For instance, Xiang et al. implemented a HIRE protocol with 80% 1RM, performed twice per week, with two sets of 8–10 repetitions per session (Xiang and Cizhong, 2017). Their findings revealed significant improvements in both whole-body and lumbar spine BMD. Similarly, Nelson et al. reported that an HIRE regimen involving two sessions per week, with three sets of 8 repetitions per session, effectively enhanced femoral neck and lumbar spine BMD (Nelson et al., 1994). These studies highlight the substantial role of HIRE in improving BMD. Specifically, HIRE promotes BMD through two physiological mechanisms. One hand, high-intensity mechanical loading activates osteoblast activity, accelerates bone formation, and suppresses osteoclast function, thereby increasing trabecular BMD and enhancing bone microstructural stability (Pagnotti et al., 2019). On the other hand, HIRE effectively strengthens muscles, further amplifying mechanical tensile forces on bones, forming a positive feedback loop that facilitates bone adaptation (Currier et al., 2023).

Recent studies have demonstrated that muscle possesses secretory functions and identified its secreted factors as myokines (Hoffmann and Weigert, 2017). These myokines exert endocrine and paracrine effects on osteoblasts, osteoclasts, and other bone metabolism-related cells, thereby modulating the dynamic balance between bone formation and resorption to either promote osteogenesis or inhibit bone degradation (Zaidi et al., 2023; Zunner et al., 2022). A growing body of experimental evidence has further supported this perspective, demonstrating the critical role of myokines in regulating the balance between osteoblasts and osteoclasts (Manske et al., 2011; Bowser et al., 2013; Zaidi et al., 2023). Notably, findings by Ali et al. revealed that HIRE induced a more pronounced myokine response compared to low-intensity, characterized by alterations in myostatin and follistatin levels, suggesting that the regulation of myokines triggered by HIRE may represent a critical mechanism underlying BMD improvements (Ataeinosrat et al., 2022).

It is noteworthy that some studies in the forest plot did not observe a positive effect of HIRE on BMD. However, this does not imply that HIRE is ineffective in improving BMD but rather underscores the necessity of optimizing its parameters. Based on network comparisons and the SUCRA analysis, the 3M (9–10) protocol ranked highest for lumbar spine and femoral neck BMD, whereas 2M (9–10) demonstrated a higher ranking for whole body BMD. Furthermore, SUCRA rankings across various skeletal sites indicate that performing 9–10 repetitions per set generally outperforms 6–8 repetitions. This may be attributed to the fact that a higher number of repetitions not only provides the bones and muscles with appropriate mechanical load and allows for sufficient recovery time but also effectively stimulates osteoblast activity while avoiding excessive osteoclast activity, resulting in better BMD improvement (Wang et al., 2022). Additionally, performing 9–10 repetitions per set significantly enhances muscle strength, thereby increasing the mechanical load applied to bones and more frequently stimulating osteoblast activity. This process promotes new bone deposition and formation in high-strain regions, ultimately exerting a positive effect on BMD (Han et al., 2023; Rajpar et al., 2023). However, further in-depth mechanistic studies are needed.

Another crucial factor that should not be overlooked is the rest interval during HIRE. In this study, the rest intervals for HIRE ranged from 60 to 180 s. Rahimi et al. investigated the acute hormonal responses to 85% 1RM HIRE with different rest intervals (60 s, 90 s, and 120 s) in young men. The results showed that shorter rest intervals (60 s) significantly increased growth hormone (GH) levels compared to longer rest intervals (120 s), whereas the increase in testosterone (TS) levels primarily occurred with the 120 s rest interval (Rahimi et al., 2010). From the perspective of BMD, the acute increase in GH may be more beneficial for bone formation, while the rise in TS levels may help reduce bone resorption. However, Schoenfeld et al. examined the effect of rest intervals on muscle hypertrophy using 75% 1RM resistance training and found that longer rest intervals (180 s) were more effective than shorter intervals (60 s) in promoting greater muscle strength and hypertrophy in resistance-trained men (Schoenfeld et al., 2016). These findings suggest that different resistance training intensities may require different rest intervals, with higher intensities potentially triggering stronger acute hormonal responses. However, as the existing studies primarily focus on young participants, direct evidence regarding whether older adults exhibit the same physiological responses is lacking.

A systematic assessment of the quality of included studies was conducted using the Rob2 and TESTEX tools. The results indicated that Item 2 in the Rob2 evaluation was consistently rated as “some concerns,” while Items 3 and 5 in the TESTEX scale mostly received a score of 0. This outcome primarily stems from the inherent challenges associated with conducting double-blind RCTs in exercise training research (Cheng et al., 2024; Noetel et al., 2024). Notably, the applicability of HIRE is constrained by individual exercise tolerance and physical fitness. Among the included studies, overall participant adherence was approximately 85%, reflecting a relatively high level of compliance. However, one study has reported an increased risk of exercise-related adverse events associated with HIRE. Specifically, Kerr et al. documented mild tendinitis in some participants following HIRE interventions, leading to a high risk of bias rating for that study (Kerr et al., 1996). These findings further underscore the necessity of individualized and progressively adjusted loading strategies in HIRE implementation to enhance adaptation in older adults and mitigate the risk of exercise-induced injuries. Additionally, the limitations of blinding procedures and the relatively small sample sizes of the included studies may contribute to potential bias. Funnel plot analysis further corroborated these findings (due to the limited number of studies on whole-body BMD (fewer than ten), the plot was not presented) (Massini et al., 2022).

Considering these factors, the overall quality of evidence in this study was rated as low according to the GRADE system. Therefore, when interpreting the findings, the potential influence of bias should be carefully considered. Future research should focus on optimizing study design, improving randomization procedures, refining blinding implementation, and enhancing control over confounding factors to reduce bias and strengthen the internal validity of findings. Additionally, while the specific details of the exercise protocols, including the type, intensity, and duration, are provided in Table 2, the lack of a specific use for exercise in our study remains an important limitation. In our study, the HIRE protocol was primarily aimed at improving BMD in elderly individuals without chronic diseases. However, based on a review of relevant literature, we found that HIRE also plays an important role in promoting muscle strength and hypertrophy, which may not have been sufficiently highlighted in the studies reviewed. Finally, as elderly individuals age, they may face an increased risk of complications such as traumatic fractures, stress injuries, and arthritis, which can reduce their tolerance to HIRE. Therefore, this study may be more applicable to elderly individuals without chronic diseases who are capable of withstanding higher exercise loads. For those with osteoporosis, diabetes, cardiovascular diseases, or other chronic conditions, it is necessary to further explore exercise programs that are safe, feasible, and well-tolerated.

## 5 Conclusion

The HIRE protocol is crucial for ensuring BMD improvement in elderly individuals without chronic diseases; however, inappropriate HIRE protocols fail to exert a positive effect on BMD. Compared to other sites, lumbar spine BMD appears to be more responsive to HIRE. HIRE with a frequency of 2–3 times per week and multiple sets appears to be the most suitable for BMD improvement, and performing 9–10 repetitions per set is particularly important for improving BMD in older adults. Furthermore, further studies on the specific mechanisms are needed, and future research should also validate these findings in practical settings.

## Data Availability

The original contributions presented in the study are included in the article/Supplementary Material, further inquiries can be directed to the corresponding author.
