# Supplementary material for: Optimization of high-intensity resistance exercise protocols for improving bone mineral density in the elderly without chronic diseases: a systematic review and network meta-analysis
Source: Front Physiol. 2025 Jun 10;16:1589200. doi: 10.3389/fphys.2025.1589200 (PMC12185277; doi:10.3389/fphys.2025.1589200)
Supplement: Supplementary file 1 [file Table1.docx]

Supplementary Table 3

Supplemental Table 3 GRADE evidence profile

| **Certainty assessment** | | | | | | | | **Effect** | **Certainty** | **Importance** |
| --- | --- | --- | --- | --- | --- | --- | --- | --- | --- | --- |
| **Studies** | **Study design** | **Risk of bias** | **Inconsistency** | **Indirectness** | **Imprecision** | **Other considerations** | **SMD (95% CI)** | |  |  |
| 10 | Lumbar Spine Bone Mineral Density | Serious^a^ | Not serious | Not serious | Serious^b^ | none | -0.07  (-0.61 to 0.46) | | ⨁⨁◯◯ Low | CRITICAL |
| 7 | Whole Body Bone Mineral Density | Serious^a^ | Not serious | Not serious | Serious^b^ | none | -0.10  (-0.42 to 0.21) | | ⨁⨁◯◯ Low | CRITICAL |
| 10 | Femoral Neck Bone Mineral Density | Serious^a^ | Not serious | Not serious | Serious^b^ | none | 0.22  (-0.23 to 0.68) | | ⨁⨁◯◯ Low | CRITICAL |

a: Implementing a double-blind randomized controlled trial for exercise interventions is challenging, which may lead to a downgrading of the risk of bias assessment.

b: Sample size below optimal information size contributing to imprecision which lowers our certainty in effect.
